# Parametric Optimization of Microcontact Stamping for Rapid Thermo-Color Change in Pigment-Coated Thin Film

**DOI:** 10.3390/mi17020238

**Published:** 2026-02-11

**Authors:** Jeonghoo Lee, Kyeongho Lee, Yeongseok Jang, Seunghoon Lee, Jinmu Jung, Jonghyun Oh

**Affiliations:** 1 Department of Nano-Bio Mechanical System Engineering, College of Engineering, Jeonbuk National University, Jeonju 54896, Republic of Korea; 2 Department of Mechanical Design Engineering, College of Engineering, Jeonbuk National University, Jeonju 54896, Republic of Korea; 3 Eyeguard Co., Ltd., Yangpyeong-ro 22-gil, Yeongdeungpo-gu, Seoul 07205, Republic of Korea

**Keywords:** thermochromism, pigment, photolithography, soft lithography, microcontact stamping

## Abstract

Microcontact stamping is a promising microfabrication technique for producing functional patterned thin films on flexible substrates; however, systematic optimization of its process parameters for thermochromic applications remains limited. In this study, we present a comprehensive parametric optimization of the microcontact stamping process to fabricate thermochromic pigment-coated thin films with rapid and reversible color responses. The effects of liquid resin type, SU-8 mold thickness, polydimethylsiloxane (PDMS) mixing ratio, and pattern size on pattern fidelity and thermochromic performance were systematically investigated. The optimal conditions were identified as a UV-curable resin, a 600 µm-thick SU-8 mold, a PDMS base-to-curing-agent ratio of 5:1, and a pattern size of 125 × 125 µm^2^. Under these conditions, the stamped thermochromic films exhibited uniform micro-patterns, rapid response and recovery behavior, and stable reversible color changes over 20 consecutive thermal cycles. This work provides practical guidelines for parameter-controlled microcontact stamping of functional thin films and demonstrates its potential for scalable fabrication of thermochromic micro-patterns. The proposed approach is expected to contribute to the development of flexible and wearable electronic devices, smart displays, and thermally responsive sensing platforms.

## 1. Introduction

Thermochromic materials exhibit temperature-dependent optical transitions that enable passive, power-free visualization of thermal stimuli. Because the colorimetric output can be read directly without external instrumentation, thermochromic coatings and films have been broadly explored for wearable sensing, human–machine interfaces, smart packaging, and adaptive thermal management platforms, where lightweight and flexible form factors are advantageous [1,2,3,4]. Among diverse thermochromic chemistries, commercially available microencapsulated leuco-dye systems remain particularly attractive for scalable manufacturing due to their tunable transition temperatures, high chromatic contrast, and compatibility with polymer matrices and coating processes [3,4,5,6,7]. However, practical deployment still faces recurring limitations: (i) durability under repeated thermal cycling, (ii) aging under UV/ambient exposure that degrades optical performance and polymer integrity, and (iii) the need to pattern thermochromic layers with controlled thickness and geometry while maintaining fast response [1,5,6,7]. Recent studies have therefore emphasized quantitative benchmarks—such as transition temperature window, optical contrast, long-term/UV stability, and mechanical retention of coated films—rather than only qualitative color-change demonstrations [1,2,5,6,7].

In parallel, rapid and spatially addressable thermochromic patterning is increasingly relevant to flexible displays and pixelated colorimetric devices, where response speed is governed by coupled heat transfer and the thermal mass of the patterned “pixel” as well as the thermal contact to the substrate [8,9,10,11]. For many thermochromic film concepts, performance trade-offs persist between optical density (often linked to film thickness and pigment loading) and switching speed, motivating process-level optimization that can control patterned thickness, area, and interfacial quality at scale [1,2,3,4,8,9,10,11]. In this context, soft-lithographic techniques offer a practical route to micro-/meso-scale patterning on polymer substrates. Microcontact printing/stamping and related elastomeric transfer methods are widely used because they are low-cost, substrate-compatible, and capable of conformal contact over large areas [12,13,14,15,16]. Recent reviews and demonstrations highlight continued progress in stamp-assisted manufacturing for functional patterns, including biomaterial and polymer systems, where throughput and uniformity are improved by controlling ink rheology, stamp mechanical properties, and contact mechanics [14,15,16,17,18].

Despite these advantages, microcontact stamping is intrinsically sensitive to the stamp and mold design parameters because elastomer deformation can distort pattern geometry or introduce incomplete transfer. Foundational and recent studies have shown that stamp deformation, roof collapse, and interfacial adhesion impose design constraints that depend on stamp modulus, feature spacing, and applied load, thereby directly influencing printing fidelity and repeatability [19,20,21]. Consequently, the mechanical properties of PDMS—strongly affected by base-to-curing-agent ratio and curing conditions—must be selected to balance conformability with dimensional stability [22,23,24]. Quantitative analyses and experimental reports indicate that deviations from standard mixing conditions can alter elastic modulus, tensile behavior, and viscoelastic response, which in turn impacts contact uniformity and pattern transfer yield for microstructured features [22,24]. Furthermore, when thick photoresist molds (e.g., SU-8) or high-aspect-ratio patterns are used, mold thickness and feature geometry become additional variables that influence stamp mechanics and contact pressure distribution, thereby requiring systematic, application-specific optimization [18,21].

The key is to use high contrast and appropriate coating technology to form letters and shapes clearly and effectively on the film. To achieve this, we present a parametric study of microcontact stamping for the rapid thermo-color change in pigment-coated thin films. Various liquids, such as UV (ultraviolet) resin, glycerol, deionized water, and mineral oil, were studied to determine the most suitable type and concentration for PDMS stamping, and the pigment powder was transformed into liquid pigments. An SU-8 mold was created on a silicon wafer using a photolithography process. The PDMS stamper was copied onto the SU-8 mold using a soft lithography process. Stamping accuracy was optimized by adjusting the thickness of the PDMS stamper and by folding and unfolding the film to evenly spread the solution on the surface. Liquid pigments that react at 35, 40, 50, and 60 °C were stamped using an optimized PDMS stamper, and performance tests were conducted to assess their sensitivity to temperature changes, demonstrating their potential application as a temperature display device on a mobile phone or computer monitor.

## 2. Materials and Methods

### 2.1. Material Preparation

To determine the optimal mixture solution for coating on the film, four solutions (deionized water, mineral oil (M8410-1L, SIGMA-ALDRICH, St. Louis, MO, USA), glycerol (G5516-500ML, SIGMA-ALDRICH, St. Louis, MO, USA), and UV resin (UV-curable adhesives, UV640, Permabond, PA, USA)) were prepared to be used as solvents to mix with the thermochromic powder. Each solvent, except for UV resin and thermochromic powder, was mixed in a 10:1 ratio and vortexed for 3 min. High-viscosity UV resin was mixed with thermochromic powder, stirred with a thin glass rod for 1 min, and finally vortexed for 5 min. We prepared four types of thermochromic pigments (Thermochromic Red 35 (TP35RE, NANO I&C Co., Asan-si, Republic of Korea), Thermochromic Red 40 (TP40RE, NANO I&C Co., Asan-si, Korea), Thermochromic Red 50 (TP50RE, NANO I&C Co., Asan-si, Republic of Korea), and Thermochromic Red 60 (TP60RE, NANO I&C Co., Asan-si, Republic of Korea)) that undergo heating and cooling cycles at 35, 40, 50, and 60 °C, respectively.

### 2.2. SU-8 Mold Fabrication

**Figure 1:**
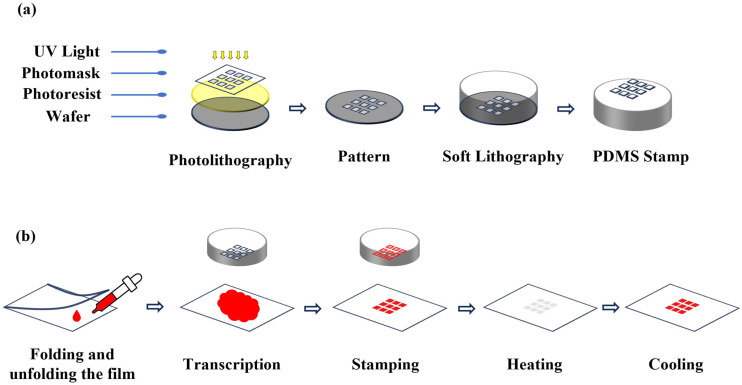
Fabrication process using (**a**) photolithography and (**b**) soft lithography.

**Figure 2:**
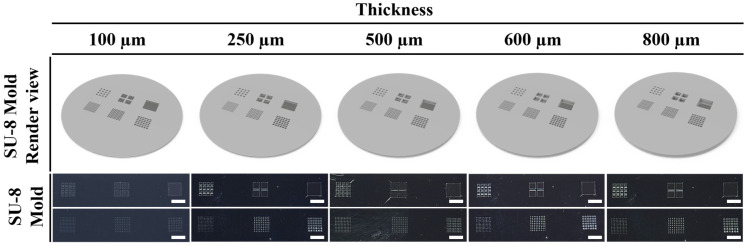
Fabricated images of SU-8 mold on a 4-inch silicon wafer with thicknesses of 100, 250, 500, 600, and 800 μm (scale bar = 5 mm).

A single film mask for a 4-inch silicon wafer, designed for use in the photolithography process, was manufactured in an array format with three different pixel sizes. Each design consisted of an 11-by-11 array with a square pixel size of 125 μm, a 9-by-9 array with a pixel size of 250 μm, and a 6-by-6 array with a pixel size of 500 μm. As shown in Figure 1a, a SU-8 mold on a silicon wafer (CZ silicon wafer, 0058-40204-001, Silicon technology corporation, Nagano, Japan) was manufactured through the photolithography process using SU-8 2100. To optimize the thickness of the PDMS stamp, patterned SU-8 molds with thicknesses of 100, 250, 500, 600, and 800 μm were fabricated, as shown in Figure 2.

### 2.3. PDMS Stamp Fabrication

First, surface modification of SU-8 was performed to facilitate separation of the SU-8 mold and PDMS. The SU-8 mold and glass slide were placed together inside a vacuum chamber, and two drops of silane solution were dropped onto the glass slide and spread evenly on the slide surface. The vacuum chamber (Vacuum Desiccator, 27-006308-05, Duran, Wertheim, Germany) was then sealed and maintained under a vacuum for approximately 1 h, allowing the silane vapor to adsorb and react on the surface of the SU-8 mold, forming a hydrophobic layer that facilitated demolding.

As shown in Figure 1b, PDMS (polydimethylsiloxane, Sylgard 184, Dow-Corning, Midland, MI, USA) stamps were fabricated using the soft lithography process. First, the polymer base and curing agent were mixed at mass ratios of 10:1, 8:1, and 5:1, respectively. The mixed solutions were degassed in a vacuum chamber for approximately 30 min to remove all air bubbles, and the debubbled mixtures were carefully poured onto a silicon master. After curing at 80 °C for 1 h in an oven (ON-02GW, W050424, JEIO TECH, Seoul, Republic of Korea), the stamps were easily separated from the mold.

Scanning electron microscopy (SEM) was employed to characterize the morphology and dimensional fidelity of the replicated PDMS pillar structures. For SEM observation, the PDMS samples were mounted on a tilted aluminum stub using conductive carbon tape to facilitate clear visualization of both lateral and vertical features. Prior to imaging, a platinum (Pt) coating with a thickness of approximately 10 nm was deposited onto the sample surface to prevent charging during electron beam exposure.

SEM imaging was performed using a Gemini 500 field-emission scanning electron microscope (Carl Zeiss Microscopy GmbH, Oberkochen, Germany) operated at an accelerating voltage of 3 kV. The lateral dimensions (width and length) and vertical height of the PDMS pillars were quantitatively analyzed from the SEM images using ImageJ software 1.54j (National Institutes of Health, Bethesda, MD, USA; https://imagej.net/ij/). For each experimental condition, five independent samples were prepared and measured to ensure statistical reliability.

### 2.4. Pattern Transfer onto Film

After applying 100 μL of each mixture to a polyethylene terephthalate (PET) film (PET film, Eyeguard, Seoul, Republic of Korea), it was thinly spread out through a folding and unfolding process using another PET film. Then, the PDMS stamp was lightly brought into contact with the corresponding area to selectively apply the mixture solution to the embossed pattern of the stamp. Afterward, this mixture solution was transferred onto the film surface as a high-resolution pattern by precisely aligning the PDMS stamp with the PET film and bringing them into contact. The patterns on the film, which were created using UV resin, were cured using a UV pen (STYLUS^®^ ATEX PENLIGHT-UV 365 nm, U271649 0518, Stremalight, Eagleville, PA, USA).

### 2.5. Thermochromic Performance Test

A film with patterns transferred using a PDMS stamp was placed on a hot plate (HP180D, MTOPS, Seoul, Republic of Korea). Temperature-dependent changes were recorded and analyzed using images and videos acquired with a stereomicroscope equipped with a digital camera featuring a 3.1-megapixel CMOS sensor and a pixel size of 2.5 µm × 2.5 µm. The mixture solution contained four different thermochromic powders that initiated color changes at 35 °C, 40 °C, 50 °C, and 60 °C, each exhibiting a common color change from red to white.

The color change video was recorded at a rate of 30 frames per second (fps), and each frame was continuously analyzed using grayscale processing to quantify temporal variations in pixel brightness. Three regions of interest (ROIs) with areas of 500 μm^2^, 250 μm^2^, and 125 μm^2^ were defined. For each ROI, pixel values corresponding to color variations were extracted and converted into grayscale intensity values. Frame indices were transformed into a time axis (1 frame ≈ 1/30 s), and the grayscale intensity values were analyzed over time to quantify the thermochromic transition from red to white. This approach enabled an objective, time-resolved evaluation of the color transition behavior induced by thermal effects.

The thermal cycling stability of the thermochromic patterns was evaluated using a microcontact-stamped film composed of a 9 × 9 pixel array with a pixel size of 250 µm. The sample was subjected to repeated thermal cycling between 25 °C and 60 °C for a total of 30 heating—cooling cycles. Temperature-dependent optical responses were recorded during each cycle.

For quantitative evaluation, six pixels were randomly selected as regions of interest (ROIs) from the patterned array. The recorded images were analyzed using ImageJ software, and the grayscale values of the selected ROIs were extracted for each heating and cooling step. The reported grayscale values represent averaged data over the selected ROIs, and the variability is expressed as standard deviation.

## 3. Results and Discussions

**Figure 3:**
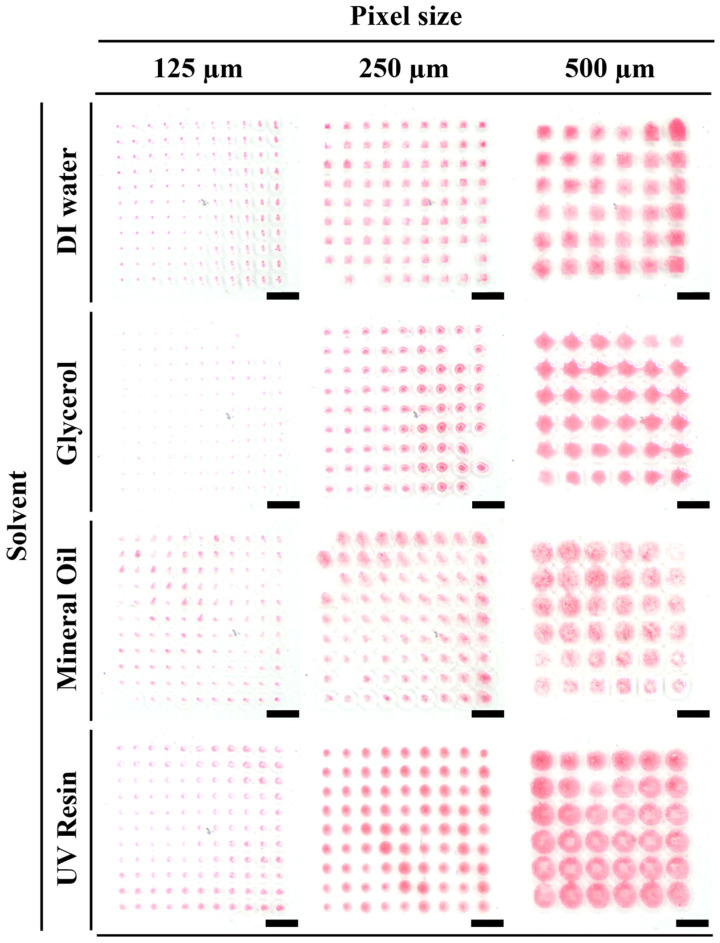
Comparison of stamping performance by pixel size for different materials (scale bar = 1 mm).

As shown in Figure 3, each pattern was transferred onto the film using four kinds of mixture solutions based on deionized water, mineral oil, glycerol, and UV resin. Patterns made with deionized water, mineral oil, and glycerol did not harden as they took a long time to dry and did not maintain the exact shape. However, patterns made using UV resin were hardened using a UV pen, so they maintained accurate shape without visible spreading over time. For this reason, UV resin was selected as the solvent for optimal stamping.

**Figure 4:**
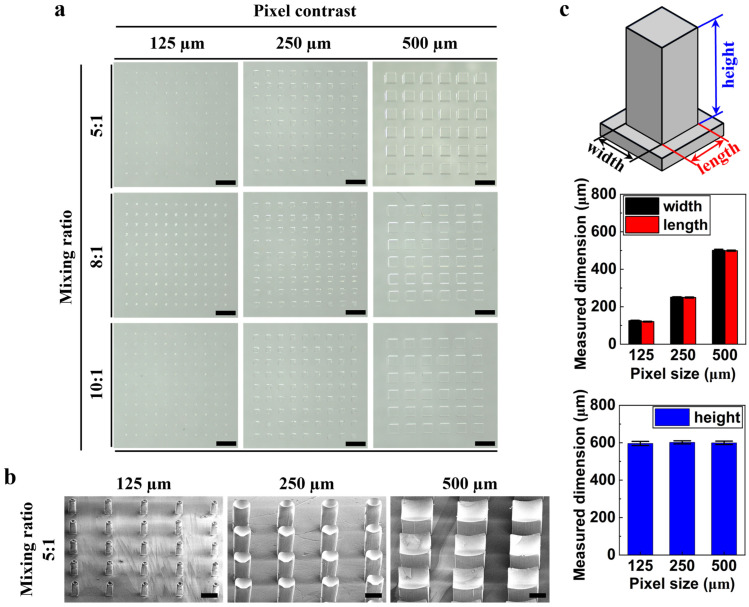
Effect of PDMS mixing ratio and pixel size on stamp fidelity and microstructure geometry. (**a**) Optical images of PDMS stamps fabricated with different PDMS base-to-curing-agent ratios and pixel sizes (scale bar = 1 mm). (**b**) Representative SEM images of PDMS pillar arrays replicated using the optimized 5:1 mixing ratio, confirming well-defined microstructures with high aspect ratios (scale bar = 200 µm). (**c**) Quantitative analysis of pillar width, length, and height extracted from SEM images as a function of pixel size, demonstrating lateral dimension scaling and consistent pillar height corresponding to the SU-8 master mold thickness.

Figure 2 shows that the SU-8 molds were manufactured with thicknesses of 100, 250, 500, 600, and 800 µm. Then, PDMS stamps were made, and stamping tests were performed. We observed that the mixture solution leaked out of the embossed pattern after stamping when thicknesses of 100, 250, and 500 µm were used. In addition, we observed that for a thickness of 800 µm, the stamp pattern was damaged due to the high aspect ratio when peeling from the mold despite the use of silane treatment. Therefore, the stamp with a thickness of 600 µm achieved the optimal stamping result. Figure 4 shows that three types of PDMS stamps were fabricated by mixing the polymer base and curing agent at mass ratios of 10:1, 8:1, and 5:1, respectively. As the polymer concentration increased at a fixed dosage of the curing agent, the mechanical strength of the PDMS stamp remained insufficient. For mixing ratios of 10:1 and 8:1, damage and structural collapse were observed on the patterned surface during the peeling process from the silicon master with high-aspect-ratio features. These results indicate that insufficient crosslinking density leads to mechanical failure when detaching the stamp from the rigid mold. Therefore, to ensure safe demolding and reliable pattern transfer, a mixing ratio of 5:1, corresponding to the highest curing agent content, was selected to fabricate mechanically robust PDMS stamps without visible damage. Figure 4b presents representative SEM images of the PDMS stamps fabricated using the optimized 5:1 mixing ratio. The images confirm that well-defined pillar arrays were successfully replicated from the silicon master, maintaining uniform geometry and high pattern fidelity across different pixel sizes. The pillars exhibit sharp edges and smooth sidewalls, indicating that the increased stiffness of the PDMS stamp effectively suppresses deformation and collapse during replication and peeling. Based on the SEM images, the lateral dimensions (width and length) and vertical height of the replicated PDMS pillars were quantitatively analyzed, and the results are summarized in Figure 4c. The measured pillar widths were 125.6 ± 2.1 µm, 251.1 ± 2.2 µm, and 501.3 ± 5.6 µm for pixel sizes of 125, 250, and 500 µm, respectively, while the corresponding pillar lengths were 124.6 ± 2.1 µm, 249.3 ± 2.9 µm, and 499.6 ± 3.1 µm. In contrast, the pillar heights remained nearly constant at 599.1 ± 2.5 µm regardless of the pixel size, which is consistent with the target thickness of the SU-8 master mold (600 µm). This result confirms that the vertical dimension of the replicated PDMS pillars is primarily governed by the mold thickness, while the lateral dimensions scale proportionally with the designed pixel size. These results demonstrate that the optimized PDMS mixing ratio enables faithful replication of high-aspect-ratio microstructures with consistent geometry, providing a mechanically stable stamp suitable for subsequent microcontact stamping of thermochromic patterns.

**Figure 5:**
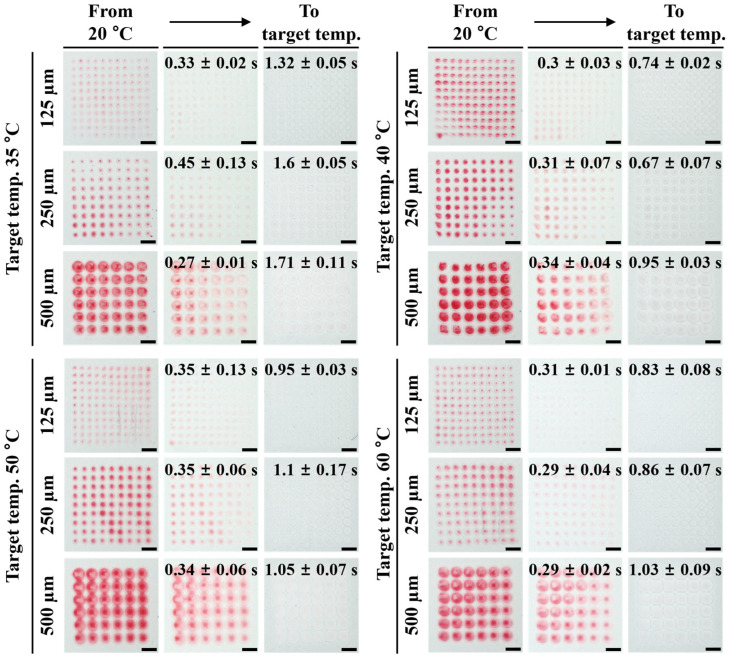
The process of the thermochromic pattern transitioning from red to white as a function of pixel size (scale bar = 1 mm).

Figure 5 shows the process of the thermochromic pattern transitioning from red to white and the reverse process. At room temperature, all patterns with pixel sizes of 125, 250, and 500 µm were red. As the temperature increased, the patterns’ colors gradually faded. At temperatures corresponding to different thermochromic heating cycles, the patterns turned completely white at 35 °C, 40 °C, 50 °C, and 60 °C. As the cooling cycles, a recovery reaction occurred, causing the patterns to revert to red, and the color change ceased at room temperature. Interestingly, the speed of reversible reactions decreased as the pattern size increased. This is because as the surface area of a pattern increases, its mass generally also increases, resulting in a greater heat capacity required to absorb or release heat. So, patterns with 125 µm pixels have a fast response, while patterns with pixels smaller than this size have poor readability. It is also worth noting that the coated pattern maintained its durability and reliability performance even after 20 repetitions of thermal cycle experiments.

**Figure 6:**
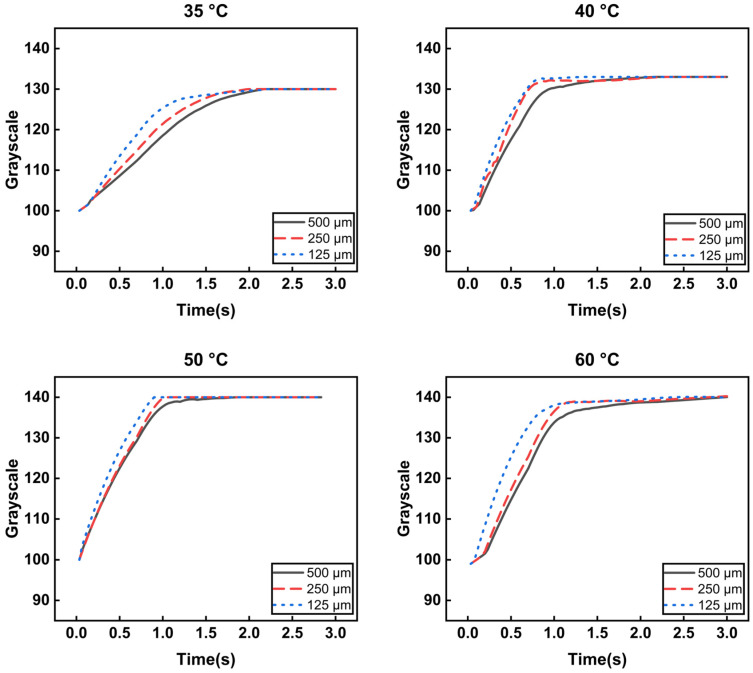
Graphs showing temperature-dependent color changes over time in a heating cycle.

The graphs in Figure 6 show temperature-dependent color changes over time in a heating cycle. The color of the patterns gradually changes from red to white as the temperature increases. The graphs of the heating cycle represent the response rate of color change from room temperature to the terminal temperature (35, 40, 50, and 60 °C) and show different behaviors depending on the size of the pattern. Figure 6 shows that the slope of the response (grayscale change/time change) decreases as the pattern size increases. As the terminal temperature of the pattern increases, its slope increases, while its response time decreases. All grayscale response curves were obtained from repeated heating–cooling experiments conducted over a total of 20 thermal cycles. The plotted curves represent averaged values, and the variability of the measurements is expressed as standard deviation, which is included in Figure 5. The relatively small standard deviations indicate that the thermochromic response behavior is highly repeatable and consistent under the tested conditions.

**Figure 7:**
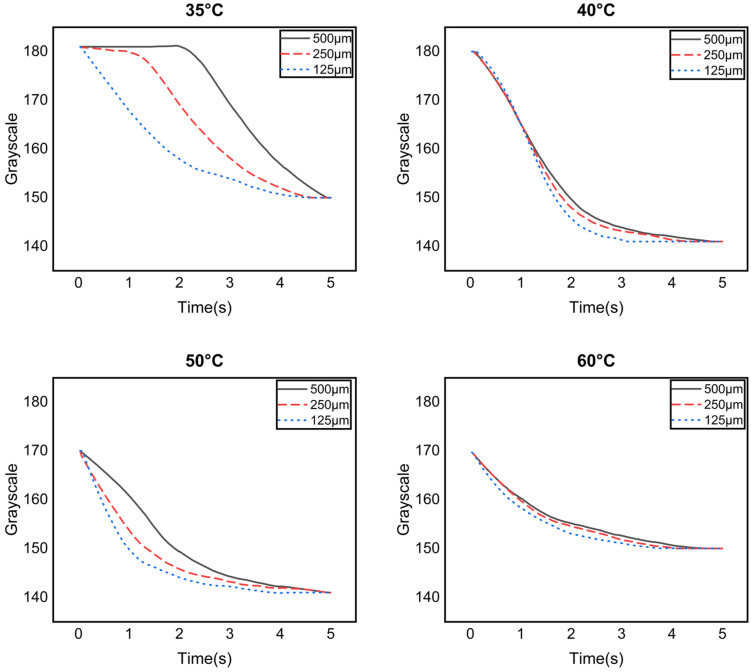
Graphs showing temperature-dependent color changes due to a cooling cycle.

The graphs in Figure 7 show temperature-dependent color changes due to a cooling cycle, where the color of the patterns gradually changes from white to red as the temperature decreases. The slopes of the graphs represent the recovery rate (grayscale change/time change) according to color changes over time. Similarly to Figure 6, it can be seen that the smaller the pattern size, the faster the recovery rate; conversely, the larger the terminal temperature, the slower the initial recovery rate.

The dependence of the thermochromic response speed on pixel size can be attributed primarily to heat transfer dynamics and differences in effective thermal mass. As the patterned area increases, both the volume of the thermochromic layer and the total heat capacity of the pixel increase, requiring a larger amount of thermal energy to reach the transition temperature during heating and to dissipate stored heat during cooling. Consequently, larger patterns exhibit slower color change and recovery behavior.

In contrast, smaller pixels possess lower thermal mass and shorter characteristic heat diffusion lengths, enabling more rapid temperature equilibration with the surrounding environment and substrate. This reduced thermal inertia allows the thermochromic material to cross its transition temperature more quickly, resulting in faster and more distinct color switching. Additionally, the increased surface-area-to-volume ratio of smaller patterns facilitates more efficient heat exchange, further accelerating the thermo-optical response.

These observations are consistent with general heat transfer principles and previously reported size-dependent thermal response behaviors in microstructured functional films. Therefore, minimizing pattern dimensions represents an effective strategy for enhancing the response speed of thermochromic micro-patterns without altering material composition.

By synthesizing the detailed results presented above, microcontact stamping can be parametrically optimized for the rapid thermochromic response of patterned pigment-coated thin films. UV resin was selected as the solvent for optimal stamping, and a PDMS stamp with a thickness of 600 µm yielded the optimal stamping result. To fabricate damage-free PDMS stamps, a 5:1 polymer-to-curing agent ratio was used. Considering the response and recovery characteristics, a pattern size of 125 µm exhibited the optimal performance.

**Figure 8:**
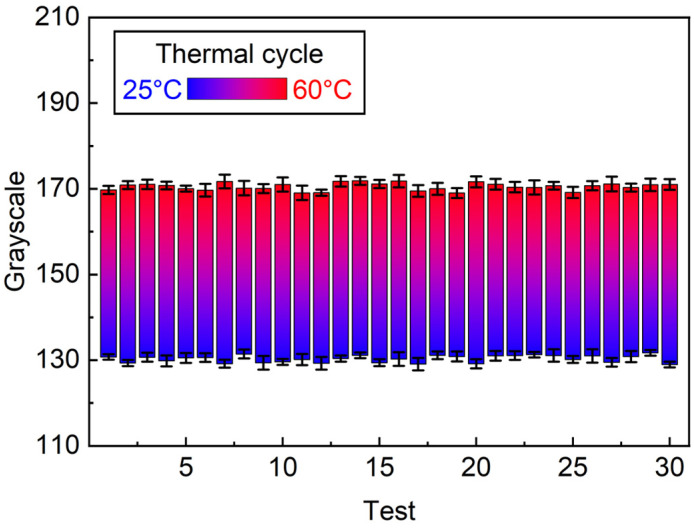
Thermal cycling stability of a thermochromic pigment-coated film patterned by microcontact stamping. Grayscale values were extracted from six randomly selected pixels in a 9 × 9 array (pixel size: 250 µm) during repeated heating–cooling cycles between 25 °C and 60 °C. Error bars represent standard deviation over 30 thermal cycles.

Figure 8 evaluates the thermal cycling stability of the thermochromic pigment-coated film patterned by microcontact stamping. The test was performed using a stamped film consisting of a 9 × 9 pixel array with a pixel size of 250 µm. The thermochromic response was repeatedly cycled between 25 °C and 60 °C for a total of 30 heating–cooling cycles. For quantitative analysis, six pixels were randomly selected as regions of interest (ROIs) from the 9 × 9 array, and their grayscale values were extracted from recorded images using ImageJ. During the heating cycles (25 °C → 60 °C), the average grayscale value remained at 170.5 ± 0.84, while during the cooling cycles (60 °C → 25 °C), the average grayscale value was 130.3 ± 0.81. The small standard deviations observed over 30 consecutive cycles indicate highly consistent and repeatable thermo-optical behavior. These results demonstrate that the thermochromic patterns maintain stable and reversible color transitions under repeated thermal cycling without noticeable signal degradation, confirming reliable operation within the tested temperature range.

The thermal cycling results shown in Figure 8 confirm the stability of the thermochromic micro-patterns fabricated by the optimized microcontact stamping process. The minimal fluctuation in grayscale values during both heating and cooling cycles indicates that the thermochromic pigment-coated film maintains consistent optical contrast and transition behavior over repeated thermal stimuli. This stability can be attributed to the uniform pigment distribution and mechanically robust pattern geometry achieved through optimized stamping parameters. The use of a moderate pixel size (250 µm) and a well-controlled PDMS stamp minimizes mechanical deformation and thermal stress accumulation during cycling, thereby preventing degradation of the thermochromic response. Furthermore, the absence of drift in grayscale values over 30 cycles suggests that the thermochromic ink and polymer matrix exhibit sufficient thermal reversibility within the tested temperature window. Although extended lifetime testing over a larger number of cycles would be required for long-term reliability assessment, the present results demonstrate stable and repeatable thermochromic performance under repeated heating–cooling conditions, supporting the applicability of the proposed stamping approach for practical thermochromic display and sensing applications.

## 4. Conclusions

In summary, the microcontact stamping process was refined through comprehensively optimizing various operational variables, including the mixture solution, SU-8 mold thickness, PDMS mixing rate, and response and recovery characteristics. UV resin was the best liquid, and the optimized stamping parameters were a 600 µm SU-8 mold thickness, a 5:1 PDMS base-to-curing agent ratio, and a 125 × 125 µm^2^ pattern size. It was also confirmed that the coated pattern exhibited stable and repeatable thermochromic performance over the tested thermal cycles, maintaining its performance through 20 consecutive cycles of reversible reactions. This parametric study for optimal microcontact stamping can be applied to arbitrary films, including PET films, and exceptionally flexible polymer substrates, catering to the requirements for advancing wearable and stretchable electronics.

## Data Availability

The original contributions presented in this study are included in the article material. Further inquiries can be directed to the corresponding authors.
